# A High Through-Put Reverse Genetic Screen Identifies Two Genes Involved in Remote Memory in Mice

**DOI:** 10.1371/journal.pone.0002121

**Published:** 2008-05-07

**Authors:** Anna Matynia, Stephan G. Anagnostaras, Brian J. Wiltgen, Maress Lacuesta, Michael S. Fanselow, Alcino J. Silva

**Affiliations:** 1 Department of Neurobiology, University of California Los Angeles, Los Angeles, California, United States of America; 2 Department of Psychiatry, University of California Los Angeles, Los Angeles, California, United States of America; 3 Department of Psychology, University of California Los Angeles, Los Angeles, California, United States of America; Centre National de la Recherche Scientifique, France

## Abstract

Previous studies have revealed that the initial stages of memory formation require several genes involved in synaptic, transcriptional and translational mechanisms. In contrast, very little is known about the molecular and cellular mechanisms underlying later stages of memory, including remote memory (i.e. 7-day memory). To identify genes required for remote memory, we screened randomly selected mouse strains harboring known mutations. In our primary reverse genetic screen, we identified 4 putative remote memory mutant strains out of a total of 54 lines analyzed. Additionally, we found 11 other mutant strains with other abnormal profiles. Secondary screens confirmed that mutations of integrin β2 (*Itg*β*2*) and steryl-O-acyl transferase 1 (*Soat1*) specifically disrupted remote memory. This study identifies some of the first genes required for remote memory, and suggests that screens of targeted mutants may be an efficient strategy to identify molecular requirements for this process.

## Introduction

Memory formation has multiple temporal phases that engage specific molecular, cellular and systems mechanisms [1], [2]. This consolidation process can also engage different structures at different stages. For example, spatial and contextual memory initially requires the hippocampus and with time engages the neocortex, a process which can take anywhere from days to weeks. Specifically in context fear conditioning, lesions of the hippocampus made one day after training lead to a complete loss of memory, whereas hippocampal lesions made 7 days after training show considerable sparing of memory[3].

In the hippocampus, learning and short-term memory formation requires synaptic molecules, such as NMDARs (N-methyl D-aspartate receptors) and CaMKII (Ca^++^ Calmodulin Kinase II), and multiple signaling pathways, such as Ras and PKA (Protein Kinase A) pathways (reviewed in[4]). These signaling pathways culminate in the activation of transcription factors (e.g. CREB (Cyclic AMP Response Element Binding protein) and C/EBPδ(CCAAT/Enhancer Binding Protein)), which lead to new RNA and proteins synthesis required for the transition from short- to long-term memory[5]. Beyond these initial stages, little is known about memory's molecular mechanisms.

Some memory, such as context fear memory, lasts a lifetime without loss [6]. However, very few studies have addressed the genetics of this persistent, “remote” memory, and to date only three mutations have been implicated in this process. First, αCaMKII heterozygous mutants (αCaMKII^+/−^) have deficits in contextual fear memory 10-, but not 1-day after training[7]. Consistent with the hypothesis that memory at 10 days is cortically dependent, the αCaMKII^+/−^ heterozygous mice have deficient cortical but normal hippocampal LTP (Long-Term Potentiation). Second, the NMDA receptor appears to also be required for remote memory, since disruption of the NR1 subunit six months following training disrupts contextual memories[8]. Third, PAK (p21-Activated Kinase), a critical regulator of actin remodeling, disrupts cortical synaptic morphology and plasticity as well as remote spatial memory[9].

Similarly, although there are numerous pharmacological manipulations that disrupt the early stages of memory, far less is known about the pharmacology of remote memory. A recent study showed that peptide inhibition of PKMξ (Protein Kinase M) in the insular cortex one month after training disrupted memory for conditioned taste aversion (CTA)[10]. Previous studies had shown that this peptide can block established CA1 LTP and memory. The results summarized above highlight the paucity of molecular and cellular information regarding remote memory.

Genetic screens have often been used as initial steps in the study of complex biological phenomena such as development, behavior and cell cycle (e.g.[11], [12], [13]). Genes identified in these screens are then used as important clues to unravel the mechanism underlying these phenomena. Previous genetic screens in mice have used ENU mutagenesis, followed by identification of the mutated gene. Although this forward genetic approach is powerful (e.g.[14]), identifying the causative mutation is still difficult and time consuming. Alternatively, reverse genetic approaches, including knockout, transgenic, and oligonucleotide interference (i.e., anti-sense RNA and RNAi), have been used to test the contribution of specific genes to biological processes of interest. However, this approach requires *a priori* knowledge often absent during the initial steps of investigating a complex biological process like remote memory.

To search for genes involved in remote memory, we designed a phenotypic screen using previously generated mutant mouse strains. This screen combines the benefits of both forward and reverse genetics, allowing for the immediate association of remote memory phenotypes with identified genes. We chose to test memory using contextual fear conditioning because the task used is quick, easily automated and much of the previous rodent work on the molecular and cellular mechanisms of memory phases used this Pavlovian task. While previously randomized screening, automated fear conditioning and remote memory have been used separately, we used these three elements in a novel synergistic combination and were able to identify two of the first genes required *specifically* for remote memory. This reverse genetic screen has the potential to identify many more remote memory mutants since less than 60 mutant strains have been screened out of more than 10,000 in public repositories (http://www.mmrrc.org/). Previous examples in the fields of development, cell cycle and more recently circadian rhythms demonstrated the power of genetic screens. Additionally, the results presented here established that remote memory is amenable to this kind of approach and demonstrated the feasibility of the novel screen that we developed.

## Results

### Primary Screen for Remote Memory Mutants

**Figure 1:**
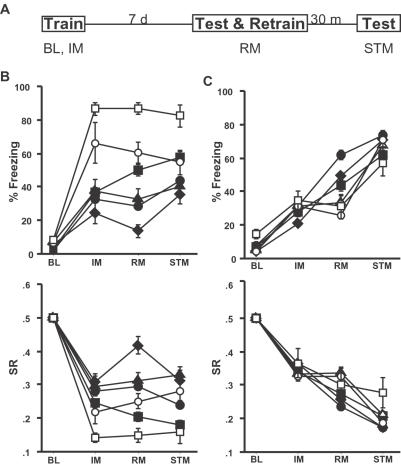
Memory profiles of different genetic backgrounds. *a*) The fear conditioning protocol used for the primary screen is depicted. Mice are trained on day 1 during which time baseline (BL) and immediate memory (IM) are assessed. Seven days later, mice are tested for remote memory (RM) and retrained in the same session. Thirty minutes later, mice are tested for short-term memory (STM). *b*) Freezing and activity suppression ratios (SR) are shown for the C57BL/6J (black circles, n = 256), C3H/HeSnJ (black diamonds, n = 8), C57BL/6NtacF (black squares, n = 67), ICR (black triangles, n = 20), Tabby (white circles, n = 6), and C57BL/6J Aw-j (white squares, n = 6) genetic backgrounds are shown for BL, IM, RM and STM time points. *c*) Freezing and activity suppression ratios (SR) are shown for mixed B6.129 (black circles, n = 262), B6.129S (black diamonds, n = 86), B6.129P (black squares, n = 60) and 129T2 (n = 29), 129S1 (n = 15) and 129P3 (n = 16) genetic backgrounds.

To identify remote memory mutants, we contextually conditioned mice and tested them 7 days later. In context conditioning, mice learn to associate the context (training chamber) with an aversive footshock. When mice were returned to the training chamber for testing, memory was assessed by measuring both the % time the mice spent freezing (lack of all movement except that for respiration) and activity suppression (the decrease in locomotor activity during the test compared to activity prior to shock) [15], [16], [17]. Freezing and activity suppression were measured prior to the shock (Baseline or BL), immediately after the shock (Immediate Memory or IM), and 7-days after training (Remote Memory or RM). At the end of the 7-day test, the mice were retrained and tested again 30 minutes later (Short-Term Memory or STM; Figure 1a); the order of testing ensured that the remote memory test was not confounded by extinction. An important consideration in screening for memory mutants is to determine if mutations disrupt performance (including perception, motivation, and motoric processes) rather than actual memory. Our primary screen controls for this important concern since the selected mutants showed normal memory in the short-term re-test (STM). Deficits in general performance would be apparent in this STM control test.

### Genetic Background Affects Memory in the Primary Screen

Since the mutant mice screened were maintained in several different genetic backgrounds, we first examined the impact of genetic background in our screen. To have an accurate representation of the memory profile for the most commonly used genetic backgrounds, we tested numerous wild type mice in different sessions. Our results were consistent with previous reports, showing that genetic background is an important factor in fear conditioning [18], [19], [20]. To adjust for the effects of genetic background on contextual fear conditioning, strains were trained with either one or three shocks (Figure 1b and c, respectively). We assigned the genetic backgrounds tested to one of these two training paradigms so as to obtain approximately equivalent wild-type freezing responses during the remote memory test (from 30–50%). This allowed for the detection of both enhancements and deficits in memory.

Even strains with similar designations can show large differences in freezing levels upon testing. For example, C57BL/6J froze less than C57BL/6NTac 7 days after training with three foot-shocks (C57BL/6J % freezing = 28.8+/−1.2, n = 256; C57BL/6NTac % freezing = 48.9+/−3.7, n = 67, F(1,321) = 51.0, p<0.0001, Figure 1b). Furthermore, two of the mutations studied were on the C57BL/6J background with the Aw-j or Tabby mutations, both of which affect coat color. The Aw-j and Tabby mutations also resulted in higher levels of freezing than the parent strain (Figure 1b). In contrast, we did not find large differences in the contextual conditioning profiles of either 129 substrains (129T2, 129S1 and 129P3) or 129B6 hybrid strains tested(129B6S and 129B6P, including F1 and F2 mice) (Figure 1c). These data highlight the importance of controlling for the *precise* genetic background in fear conditioning studies [21].

### Validation of the Remote Memory Screen

**Figure 2:**
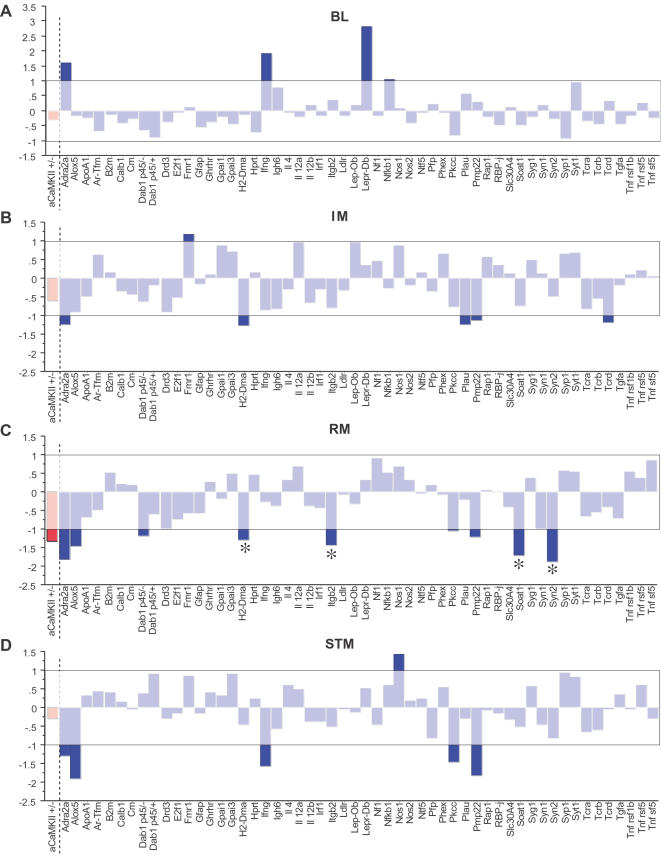
Primary screening of 54 mutant strains identifies 4 putative remote memory mutants. Combined mean z-scores for freezing and activity suppression ratios are shown for all 54 mutant strains for *a*) baseline, *b*) immediate memory, *c*) remote memory and *d*) short-term memory results. The white shaded box denotes values that fall within +/−1.0 standard deviation from the normal population. Mean z-scores for the αCaMKII^+/−^ mutant mice (see also Supplemental Figure S1 and Text S1) are shown in red for comparison. The asterisks denote putative remote memory mutants. BL = Baseline; IM = immediate memory; RM = 7 day memory; STM = short-term memory.

To determine the effectiveness of our primary screen, we tested the only mouse mutant strain (αCaMKII^+/−^) known to show dramatic deficits in remote, but not in short-term, memory. Consistent with previous studies[7], [22], the αCaMKII^+/−^ **hetero**zygous mutants show nearly normal short-term (STM test), but profoundly deficient remote (RM test), memory (Figure 2, red bar). To further validate the screen we tested a number of other manipulations that are known to affect specific memory phases (Supplemental Figure S1 and Text S1). Taken together, these data show that our primary screen can identify mutants with normal short-term memory, but deficient remote memory.

### Standardization of the Primary Screen Results

Each mutant strain tested was compared to both the small group of wild-type controls trained and tested at the same time, and to the large group of wild-type mice of the same genetic background. This way, we obtained both individual and population-based comparisons. To facilitate population-based comparisons, we used standard (z) scores, which allowed us to plot mice from different genetic backgrounds and training regiments on the same statistical scale. Z-scores also allowed us to combine both freezing and activity scores collected for the same mice (see Methods). Since memory deficits should result in corollary alterations in both freezing and activity suppression, combining z-scores for both of these measures simplified comparisons and improved reliability.

### Memory Mutants Identified in the Primary Screen

In our primary screen, 54 previously generated mutant mouse strains were tested, including 42 knockout mutants, 9 point-mutants, 2 transgenic dominant-negative mutants and 1 mutant with a chromosomal deletion. Forty-four of these were randomly selected using a random-number generator to select catalogue numbers from the Jackson Laboratories Genetically Engineered and Mutant Mice Resource. The remaining 10 were obtained from other laboratories (see Table 1). For each strain, we compared approximately 8 mutants to 8 wild types of the same genetic background in the same training session. A summary of z-scores for Baseline (Figure 2a), Immediate (Figure 2b), Remote (Figure 2c) and Short-Term Memory tests (Figure 2d) is shown. For reference, we have shown the combined z-scores for the αCaMKII^+/−^ mice used to validate the screen. These mutants displayed a z-score between 1.0 and −1.0 for both the immediate and short-term memory tests, but showed z scores lower than this cut off point in the remote memory test, a result consistent with their remote memory deficits [7]. Accordingly, we selected mutant strains for secondary screens with z-scores profiles similar to αCaMKII^+/−^ mice.

**Table 1:** Fifty-four mutant strains were tested in the primary screen.

strain name	full name	MGI accession #	Catalogue/Reference
αCaMKII^ΔEx2^	α Ca^++^ Calmodulin Kinase II, exon 2 deletion	MGI:88256	[^48^]
Adra2a	adrenergic receptor α2a	MGI:87934	2777
Alox5	arachidonate 5-lipoxygenase	MGI:87999	2263
ApoA1	apolipoprotein A1	MGI:88049	2055
Ar-Tfm	androgen receptor-testicular feminization	MGI:88064	1809
B2m	beta-2 microglobulin	MGI:88127	2087
Calb1	calbindin-D28K	MGI:88248	3079
Cm	coloboma	MGI:88424	1547
Dab1	disabled 1 (p45/− and p45/− alleles)	MGI:108554	[49]
Drd3	dopamine receptor D3	MGI:94925	2958
E2f1	E2F transcription factor 1	MGI:101941	2785
Fmr1	fragile X mental retardation syndrome 1	MGI:95564	3025
Gfap	glial fibrillary acidic protein	MGI:95697	2642
Ghrhr	growth hormone releasing hormone receptor	MGI:95710	533
Gpai1	G protein αi1	MGI:95771	[^50^]
Gpai3	G protein αi3	MGI:95773	[^50^]
H2-Dma	histocompatibility 2, class II, locus DMa	MGI:95921	2643
Hprt	hypoxanthine phosphoribosyltransgerase	MGI:96217	2171
Ifng	interferon γ	MGI:107656	2287
Igh-6	immunoglobulin heavy chain-6 (heavy chain of IgM)	MGI:96448	2288
IL 4	interleukin 4	MGI:96556	2253
Il 12a	interleukin 12a	MGI:96539	2692
Il 12b	interleukin 12b	MGI:96540	2693
Irf-1	interferon regulatory factor 1	MGI:96590	2762
Itgβ2	integrin β2	MGI:96611	2128
Ldlr	low density lipoprotein receptor	MGI:96765	2207
Lep-Ob	leptin	MGI:104663	632
Lepr-Db	leptin receptor	MGI:104993	697
Nf1	neurofibromatosis 1	MGI:97306	[51]
Nfκb	nuclear factor of κ light chain gene enhancer in B-cells 1, p105	MGI:97312	2849
Nos1	nitric oxide synthase 1, neuronal	MGI:97360	2633
Nos2	nitric oxide synthase 2, inducible, macrophage	MGI:97361	2609
Ntf5	neurotrophin-5	MGI:97381	2497
Pfp	pore-forming protein	MGI:97551	2407
Phex	phosphate regulating gene with homologies to endopeptidases on the X chromosome	MGI:107489	528
Pkcc	protein kinase C, γ	MGI:97597	2466
Plau	plasminogen activator, urokinase	MGI:97611	2509
Pmp22Tr-J	peripheral myelin protein, 22 kDa	MGI:97631	2504
Rap1	Ras-related protein 1	MGI:894315	[52]
RBP-j	recombination signal binding protein for Igg κ J region	MGI:96522	Silva*
Slc30a4	solute carrier family 30 (zinc transporter), member 4	MGI:1345282	219
Soat1	sterol O-acyltransferase 1	MGI:104665	2896
Syg1	synaptogyrin 1	MGI:1328323	[^53^]
Syn1	synapsin 1	MGI:98460	[^54^]
Syn2	synapsin 2	MGI:103020	2477
Syp1	synaptophysin 1	MGI:99667	[^55^]
Syt1	synaptotagmin 1	MGI:99667	[^56^]
Tcr a	T cell receptor α	MGI:98553	2116
Tcr b	T cell receptor β	MGI:98578	2118
Tcr d	T cell receptor δ	MGI:98611	2120
Tgfa	transforming growth factor α	MGI:98724	2219
Tnf rsf1b	tumor necrosis factor receptor superfamily member 1b	MGI:1314883	2620
Tnf rsf5	tumor necrosis factor receptor superfamily member 5	MGI:88336	2928
Tnf sf5	tumor necrosis factor superfamily member 5	MGI:88337	2770

* Unpublished dominant negative mutant mouse strain, A.J. Silva.

The strain name, mutated gene, MGI accession number and Jackson Laboratories catalogue number or reference for each of the mutant strains tested in the primary screen is listed.

**Figure 3:**
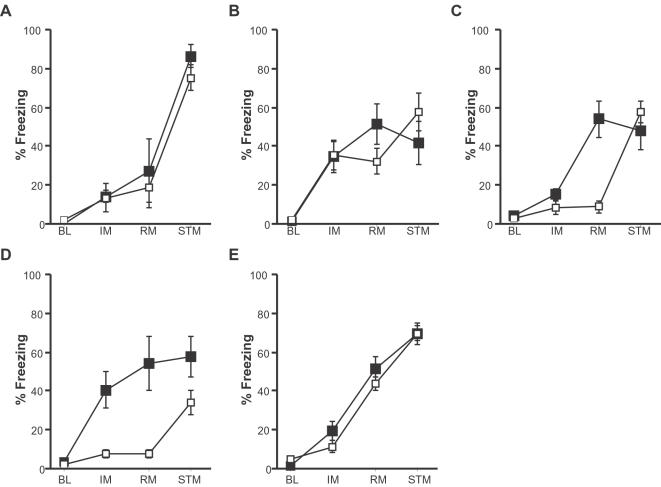
Verification of primary screen confirms three putative remote memory mutant strains. *a*) Dab1^p45/−^ mice (white) show normal memory compared to their controls, Dab1^p45/+^ (black) at all times tested. *b*) Itgb2^−/−^ mice showed a trend towards decreased freezing in the RM test. *c*) Soat1^−/−^ mice specifically exhibit RM deficits. *d*) Syn2^−/−^ mice show a deficit in both the IM and RM tests. *e*) H2-DMa^−/−^ mice exhibit normal memory at all times tested. BL = Baseline; IM = immediate memory; RM = 7 day memory; STM = short-term memory.

Sixteen of the 54 mutant strains screened revealed changes in at least one of the measures taken: two mutant strains showed enhanced baseline freezing (Figure 2a), four showed deficits or enhancements in immediate or short-term freezing (Figure 2b and d), two revealed impairments in both remote and short-term memory and the last three exhibited deficits in another combination of tests (Figure 2a,b,c and d). Of the 54 mutant strains screened, H2-Dma^−/−^, Itgβ2^−/−^, Soat1^−/−^ and Syn2^−/−^ showed relatively specific 7-day memory deficits. Interestingly, although the Dab1^p45/−^ mice showed a remote memory deficit in our population based comparison, there were no differences when compared to their littermate controls and therefore we did not pursue their study (Figure 3a).

### Secondary screens

As in all genetic screens, primary screens represent a rough first pass through a large number of mutants so that the bulk of the analysis (secondary screens) can be focused on the most promising candidates. For the studies described in this section, we used the same breeding strategy as described by the vendor (see methods). First, a second set of mutants and their controls bred in house was tested using the primary screen protocol. Transportation and dramatic changes in housing conditions and routines can have a profound impact on rodent behavior, including behaviors known to depend on emotional systems, such as fear conditioning. Comparisons with their wild type controls show that the remote memory deficit was again observed in the Itgβ2^−/−^, Soat1^−/−^ and Syn2^−/−^ mice (Figure 3b, c and d, respectively) and that they had normal STM. However, we were unable to replicate the remote memory deficit in the H2-Dma^−/−^ mice, (Figure 3e), suggesting that the original results represent either a false positive, or experience-dependent effects (e.g. from mouse transportation).

**Figure 4:**
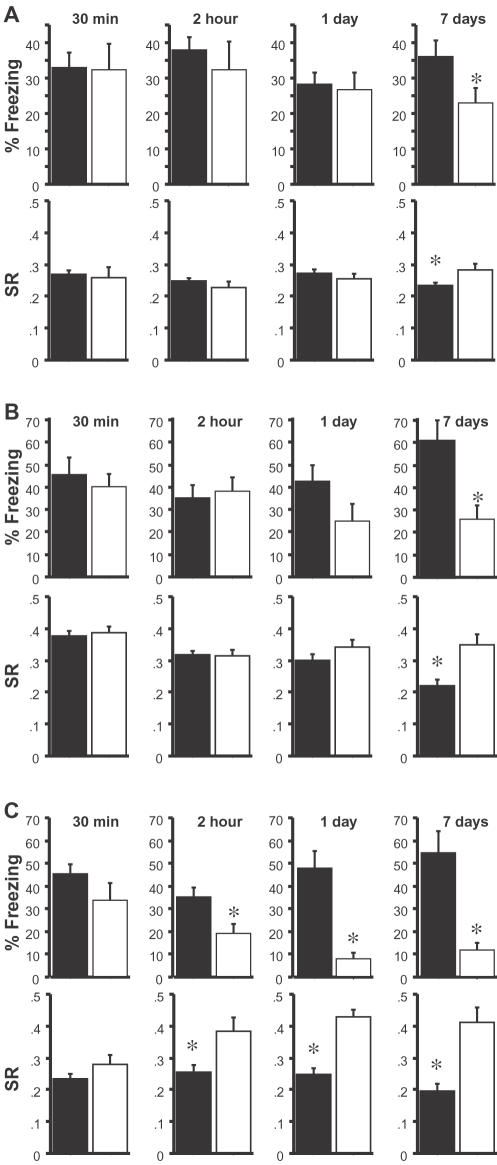
Secondary screening identifies two remote memory mutant strains. Freezing and activity suppression ratios (SR) are shown. *a*) Itgb2^−/−^ mutant mice (white) show normal memory at 30 minutes (F_1,24_ = 0.011, p = 0.92; wt, n = 16; mutant, n = 10), 2 hours (F_1,22_ = 0.41, p = 0.53; wt, n = 12; mutant, n = 12), and 1 day (F_1,34_ = 0.092, p = 0.76; wt, n = 23; mutant, n = 13) compared to wild type controls (black). These mice show a deficit at 7 days (F_1,33_ = 4.65, p = 0.039; wt, n = 20; mutant, n = 15). Both Itgb2^−/−^ and wild type mice exhibited 0–6% baseline freezing for each time point. *b*) Soat1^−/−^ mutant mice (white) exhibit normal memory at 30 minutes (F_1,26_ = 0.31, p = 0.58; wt, n = 15; mutant, n = 13), 2 hours (F_1,23_ = 0.16, p = 0.69; wt, n = 13; mutant, n = 12) and 1 day (F_1,22_ = 2.99, p = 0.098; wt, n = 13; mutant, n = 11) compared to wild type controls (black). However, Soat1^−/−^ mice exhibit a statistically significant deficit at 7 days (F_1,14_ = 10.3, p = 0.006; wt, n = 8; mutant, n = 8). Both Soat1^−/−^ and wild type mice exhibited 2–9% baseline freezing for each time point. *c*) Syn2^−/−^ mutant mice (white) show normal memory only at 30 minutes (F_1,18_ = 1.577, p = 0.225; wt, n = 15; mutant, n = 13) compared to wild type controls (black). Syn2^−/−^ mice show a deficit at 2 hours (F_1,18_ = 7.00, p = 0.016; wt, n = 10; mutant, n = 10), 1 day (F_1,23_ = 23.5, p<0.0001; wt, n = 12; mutant, n = 13) and 7 days (F_1,18_ = 22.6, p = 0.0002; wt, n = 9; mutant, n = 11). Both Syn2^−/−^ and wild type mice exhibited 3–5% baseline freezing for each time point.

We next examined in more detail the time course of memory loss in the Itgβ2^−/−^, Soat1^−/−^, and Syn2^−/−^ mutants. Separate groups of mice were tested at 30 minutes, 2 hours, 1-day and 7-days after contextual fear conditioning training. These time points were selected to confirm the 30-minute and 7-day data from previous tests and to further differentiate between mutations affecting initial stages of memory (memory tested at 2 hours and 1 day) and those changing remote memory (7-day test). As before, training protocols were dependent on genetic background: the Itgβ2^−/−^ and Syn2^−/−^ mice (C57BL/6J genetic background) were trained with the 3-shock protocol, while the Soat1^−/−^ mice (129B6S genetic background) were trained with the 1-shock-protocol. Itgβ2^−/−^ mice had intact contextual memory at 30 min, 2 h and 1 day, but significantly impaired memory at 7 days (Figure 4a). As suggested by the data in the two previous tests, the remote memory deficit was moderate but significant. Similarly, Soat1^−/−^ mice had intact memory at 30 min and 2 h, but not at 7 days (Figure 4b). Although there was a trend towards decreased memory at 1-day in these mice, this did not reach statistical significance (p = 0.1 with n = 13 wild-type; n = 11 mutant). In contrast, detailed analysis of their memory deficits showed that the Syn2^−/−^ mice had intact memory at 30-min, but abnormal memory at 2-hour, 1-day and 7-days (Figure 4c). Indeed, these mutants were previously shown to have a 1-day memory deficit in fear conditioning[23]. Because the mutants showed small differences in baseline activity (see Supplemental Table S1), we also analyzed another measure of fear conditioning that minimizes the effect of baseline activity differences (activity suppression): the results confirmed the findings obtained using freezing scores (Figure 4a, b and c). These findings confirm the remote memory deficits in both the Itgβ2^−/−^ and Soat1^−/−^ mice, and they attest to the efficacy of our remote memory screen.

### Impact of genetic background

Besides studying the Itgβ2^−/−^ and Soat1^−/−^ mutants in the genetic background obtained from the vendor, we also bred the Itgβ2^−/−^ and Soat1^−/−^ mice into the C57BL/6J and C57BL/6NTac, respectively. Potential changes in phenotypes may allow future identification of remote memory genetic modifiers. During this backcrossing process (Supplemental methods Text S2), we tested again the 7-day memory phenotype of both mutants. The results showed that changing the genetic background of the Itgβ2^−/−^ and Soat1^−/−^ mutations rescued their memory phenotype (Supplemental Figure S2 and Text S3), a result that adds to the emerging body of evidence that behavior, just like every other biological phenotype, is very sensitive to modifiers in the genetic background. Specifically, these findings show that genetic modifiers in C57BL/6J and C57BL/6NTac interact with the Itgβ2^−/−^ and Soat1^−/−^ respectively, to rescue their remote memory deficits.

### Remote Memory Deficits are Specific to Hippocampus-Cortex Dependent Memory

**Figure 5:**
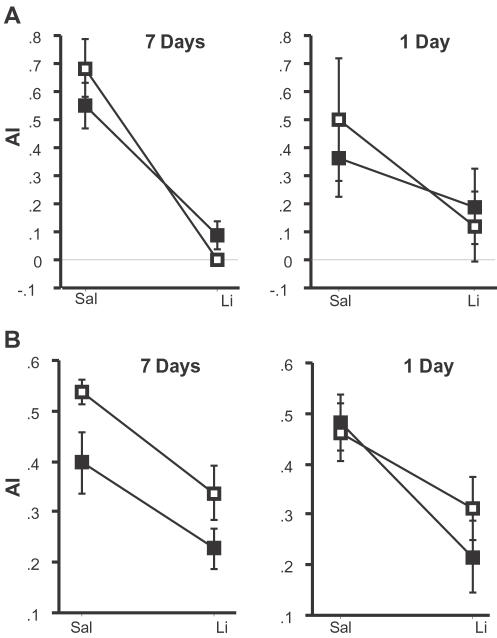
Itgb2^−/−^ and Soat1^−/−^ mice have normal memory in conditioned taste aversion (CTA). The aversion index (AI) to saline (Sal)- or lithium chloride (Li)-paired flavored food is shown for both mutants. *a*) Itgb2^−/−^ mice (white) show normal 7-day (F_1,40_ = 2.65, p = 0.11) and 1-day (F_1,44_ = 2.04, p = 0.16) memory for the Li-paired food compared to wild type controls (black). *b*) Soat1^−/−^ mice (white) also exhibit normal memory at 7-days (F_1,47_ = 0.082, p = 0.78) and 1-day (F_1,49_ = 0.91, p = 0.34) for the Li-paired food compared to their wild type controls (black).

To determine whether the Itgβ2^−/−^ and Soat1^−/−^ mutations (in the original genetic background) affected other forms of memory, we also tested these mutants in conditioned taste aversion (CTA). Unlike contextual conditioning, CTA does not appear to have either a temporal gradient or hippocampus-dependency[24], [25]. In this memory test, a novelly-flavored food is paired with a malaise-inducing agent (lithium chloride), and memory of this association is shown by aversion to the flavored food. For the studies described in this section, we used the same breeding strategy as used in the section describing our secondary screen (see methods). Again, as the two mutants tested are maintained in different genetic backgrounds, the wild type mice display somewhat different initial preferences for the flavored food (Figure 5). In tests given 7 days after training, both Itgβ2^−/−^ (Figure 5a) and Soat1^−/−^ (Figure 5b) showed aversion to the flavored food paired with lithium chloride but no aversion to the food paired with saline (control). The same animals were later trained with a different flavored food and tested 1 day later. Once again, neither mutation affected memory for CTA. Since this form of memory is known to be dependent on a number of brain structures, including gustatory cortex and amygdala [24], [25], [26], these results attest to the specificity of the remote memory deficits of these two mutant strains.

## Discussion

Early memory phases, including immediate and short-term memory, are defined by unique molecular, cellular and system processes. By comparison little is known about late memory phases, including remote memory. Here, we devised a novel genetic screen that combines the advantages of both forward and reverse genetic approaches, and identified two new genes required for remote memory: integrin beta2 and steryl-O-acyl transferase 1. Importantly, our studies also demonstrated the feasibility of this approach since two mutants were found out of a total pool of 54 tested. It is estimated that there are 10,000 other mutant mice available from a number of international repositories (http://www.mmrrc.org/), which would suggest that our screen could reveal a considerable number of additional genes required for remote memory. Additionally, we also uncovered evidence for genetic modifiers for remote memory mutations. Altogether, these genetic tools will undoubtedly be critical for the study of the molecular, cellular and systems underpinnings of this process.

The search for genes involved in memory began with a screen in fruit flies, ultimately determining that the cAMP signaling pathway is an integral component of memory formation [2]. Since then studies in Drosphila [12], aplysia [27] and rodents [4] implicated a number of other genes in memory. To date, most of these studies have focused on mechanisms of memory acquisition and protein synthesis-dependent consolidation – events that are completed within the first few hours following acquisition. However, additional processing/maintenance of memory occurs after this initial period. For example, hippocampal memories (i.e. contextual conditioning) become gradually cortically-based [1], [28]. The results presented here demonstrate that integrin beta2 and steryl-O-acyl transferase 1 are required for processes that occur well beyond the initial acquisition and initial consolidation of memory, since the mutations that we identified affect 7-, but not 1-day memory.

Mice with knockout mutations, point mutations, chromosomal deletions and constitutive or regulated overexpression of transgenes were randomly selected and subjected to our primary screen. Our reverse genetic screen is not meant to substitute for detailed analysis of targeted mutations. Instead, it is similar to forward genetic approaches, including those that used the chemical mutagen ENU in searches for novel learning and memory loci [29], [30], [31]. In both approaches, no a priori assumptions are made about the nature of the genes to be discovered or the mechanisms that connect them to the relevant phenotypes; both forward and reverse genetic screens begin with primary tests designed to weed out unrelated mutants, followed by a more extensive analysis of candidate mutants with a set of secondary tests. However, key differences confer each approach with advantages and disadvantages. For example, reverse genetic screens are limited by the mutants available for screening, while forward genetic screens can theoretically scan every gene in the genome. In contrast, the process of gene identification is far more laborious in forward genetic screens since a causative (usually point) mutation must be identified amongst the entire genome.

In our screen, we used contextual fear conditioning to identify genetic alterations in mice that affect remote memory. In addition to the individual mutant strains tested, our database includes a large number of wild type mice that provided an accurate description of normal distributions of the behavioral measures included in our screen. This wild type data set was useful in choosing which mutants to pursue in our secondary screen. For example, a considerable percentage of wild type mice (6–16%) in each of the genetic backgrounds failed to freeze in the 7-day test; therefore, in a group of eight mice with normal remote memory, only one or two mice would be expected to have a z-score outside of +/−1 standard deviation. A mutant strain for which the group of eight mice have a mean z-score outside of +/−1 is an ideal candidate for further analysis. Thus, we used these z-score limits as the thresholds to select strains to follow up in our secondary screens. This control data set also demonstrates the critical importance of genetic background in behavioral phenotypes, a point that has been highlighted in numerous previous studies [18], [19], [32], [33]. Indeed, our results emphasize this important fact, since not only did we find differences in performance between various wild type genetic backgrounds, we also saw dramatic phenotypic differences in the mutant strains carrying the same mutation but in a different genetic background as analyzed in detailed secondary screens. As we changed the genetic background of these two strains, their associated remote memory phenotypes also changed. This will provide an invaluable opportunity to clone loci that modify the function of the two genes identified in our screen.

To increase the specificity of the primary screen, we included two key controls: immediate and short-term memory tests. These controls allowed us to eliminate mutants with performance deficits (i.e. unable to show freezing responses) since such mutant strains would have resulted in deficits at all 3 time points tested. In our primary screen, four potential remote memory mutants were identified. Subsequent testing showed that three of these mutants had reproducible deficits, but one strain, H2-Dma^−/−^, had normal remote memory. The reason for this false positive is unknown but could be due to a number of factors, including the small number of mice tested in the primary screen. Consequently, data from primary screens is always verified and further explored in subsequent tests.

In addition to remote memory mutants, our primary screen identified several other potential categories of mutants. For example, we identified 5 mutations that showed normal immediate memory, but abnormal short-term memory, the memory profile we obtained in our primary screen for mice with hippocampal lesions. Although there are a number of possible explanations for this memory profile, it is possible that this class of mutants is enriched for mice with abnormal hippocampal function. In addition to loss of function mutants, our primary screen also detected mutant mice with seemingly enhanced memory in immediate or short-term memory. However, these phenotypes were not the primary target of our screen and therefore, they have not been verified or tested in secondary screens.

To characterize further the memory profile of the remote mutants identified in the primary screen, we examined the time course of memory loss of Itgβ2^−/−^, Soat1^−/−^ and Syn2^−/−^ mutants (secondary screen). The results demonstrated that both the Itgβ2 and Soat1 strains have intact immediate memory and short-term (30 min or 2 h) as well as intact memory after the protein synthesis-dependent window (tested at one-day), but abnormal remote (seven-day) memory. These data indicate that events involved in the acquisition and protein synthesis-dependent memory consolidation occur normally, ruling out alterations in a number of hippocampal processes ranging from synaptic signaling to transcription/translation. Furthermore, the remote memory deficits are likely to be specific to cortical consolidation of hippocampal–dependent memories since 7-day memory for CTA was normal for both mutants. Accordingly, previous studies suggested that remote memory for contextual fear conditioning and CTA involve different cortical regions [22], [25]. Similar to the αCaMKII^+/−^ **hetero**zygous mutation, the Itgβ2^−/−^ and Soat1^−/−^ mutations may affect physiological processes (i.e. temporal cortex LTP) required for remote memory without disrupting hippocampal-dependent memory formation [3], [7], [34], [35]. Alternatively, *Itg*β*2* and *Soat1* could be involved in other processes associated with later phases of memory consolidation, such as synaptic restructuring[36], [37].

Soat1 is a critical enzyme in cholesterol metabolism. The role of cholesterol at the synapse is multifaceted: it is specifically required for synapse formation in neuronal cultures[38], for syntaxin I clustering[39] which is required for synaptic vesicle fusion and it may otherwise influence synaptic vesicles by binding the abundant synaptic vesicle proteins, synaptotagmin I and synaptophysin[40]. Since cholesterol cannot cross the blood-brain barrier, all CNS cholesterol is from *de novo* synthesis requiring intact and active cholesterol metabolic machinery. Furthermore, deficits in cholesterol metabolism are often associated with mental retardation (reviewed in[41],[42]), suggesting that cholesterol metabolism is crucial for normal brain development and function. Additionally, Soat1 has been associated with aging in microarray studies and with Alzheimer's in genetic studies[43], [44]. Importantly, the Soat1 mice learnt and had normal 1-day memory suggesting that they had no major developmental defects. The deletion of Soat1 specifically causes memory impairment at remote time points.

Integrin β2^−/−^ mice also showed a remote memory deficit. Integrin β2 (CD18, LFA-1) is localized on leukocytes and, as a heterodimer, binds various ICAMs (IntraCellular Adhesion Molecules). This molecule has been examined in the context of CNS injury. Integrin β2 has a critical role in the phagocytosis of injured neurons[45]. It is possible that in the absence of injury, Integrin β2 may mediate normal remodeling of neuronal structure important for remote memory. Integrin β2 and ICAM1 are also expressed on activated microglia found in the vicinity of amyloid deposits in Alzheimer's disease. Interestingly, *Itg*β*2* is located near the breakpoint for trisomy 21 and could thus contribute to Down's Syndrome ([46] but see[47]). Taken together, these data indicate a possible contribution of integrin β2 to normal cognitive function via structural remodeling or pruning, two processes that may be required for remote memory.

The results of this study mark the beginning of a systematic genetic dissection of remote memory. We identified two novel remote memory mutants with very diverse cellular roles, Itgβ2^−/−^ and Soat1^−/−^. Both of these mutants have intact memory acquisition and protein synthesis-dependent consolidation, but showed reproducible deficits in three separate remote memory tests. The approach we developed has the potential to identify many other novel genes since we only screened less than 1% of the current mouse mutant resource. These genes will be valuable tools to elucidate the molecular, cellular and systems processes underlying remote memory.

## Materials and Methods

### Mice

Wild type mice of different genetic backgrounds were purchased from Taconic Farms (Germantown, NY) or Jackson Laboratories (Bar Harbor, ME). To select mutant mice in an unbiased manner, a random number generator was used to obtain catalogue numbers from the Jackson Laboratories. All mice were group housed, maintained in a 12∶12 light/dark cycle and had water and food ad libitum. All experiments were performed in accordance with the institutional guidelines of the University of California at Los Angeles. Mutant strains that showed a 7-day memory deficit in the primary screen (and their controls) were bred according to the Jackson Laboratories protocol for each mutant strain. See supplemental methods (Text S2) for back-crossing.

### Primary screen protocol

Wherever possible, eight mutants and 8 controls were subjected to an 8 minute training session with 1 tone–shock pairing at 2 minutes for 129;B6 and 129 backgrounds or 3 tone-shock pairings at 2, 3 and 4 minutes for C57BL/6 and C3H backgrounds. The 129;B6 background included 129;B6S F1, 129;B6S F2, 129;B6P F1 and 129;B6S F2 mice and the 129Sv background included 129P3, 129S1 and 129T2 mice, according to the Jackson nomenclature. Tone–shock pairs consisted of a 30-second tone that co-terminated with a 2 second 0.75 mA constant current scrambled shock. Minutes 0–2 were used to measure baseline freezing. Immediate freezing was assessed from minutes 6–8. After 7 days, the mice were returned to the same chamber for a 5 minute session in which the first 2 minutes were used to assess remote contextual memory and a single tone/shock pair was delivered at minute 4. The mice were returned to their home cage for 30 minutes and then re-tested for short-term memory as described above but without the foot-shock. Two-minute time intervals were used for all tests so that they could be directly compared across time points and across strains. Data for tone tests is not shown.

### Secondary screen protocol

Separate groups of mice (at least 8 mutant and 8 wild types per time point) were trained as described for the primary screen, and tested at 30 min, 2 hours, 1 day or 7 days. The context test consisted of a 5-minute session in the training chamber during which memory was assessed.

### Conditioned Taste Aversion

Mice were food deprived to 90% of their free-feeding body weight then habituated in individual cages to eat control chow pellets from ceramic cups for 3 days. On the training day, mice were given 20 chocolate or sucrose (counter balanced) pellets and then allowed to feed for 45 minutes at which time, 2% body weight of 0.15 M LiCl or 0.15 M NaCl (control) was administered by ip injection. Seven days later, the mice were tested by giving a choice between 20 flavored food pellets or 20 control chow pellets. After a three day interval, the same mice were re-trained for the 1 day test, switching both the food flavor and the injected agent such that each mouse receive lithium chloride once and each novel flavor only once. The aversion index was calculated as (# of pellets of novel food eaten)/(total number of pellets eaten).

### Data analysis

Automated freezing (% FR) and activity scores (arbitrary units) were calculated using a previously described computer algorithm[17]. Suppression of activity ratios (SR) were calculated as (test activity)/(test+baseline activity), which normalizes for hyper- or hypo-activity. Z-scores for the entire wild type population for each genetic background were normalized for each test using the equation (individual-population mean)/standard deviation. Thus, for any given score in any given background, the expected value for each genetic background is a mean of 0 and a standard deviation of 1. Values for freezing and activity suppression were converted to z-scores for each mutant and their wildtype controls using the equation z-score = average[(%FR individual–%FR population mean)/(standard deviation of population), (−1)*(SR individual–SR population mean)/(standard deviation of the population)], using the population scores for the appropriate genetic background. The z-score for activity suppression was inverted so that it would be on the same scale as freezing. Thus, for any given test, deviations from the expected value of 0 could be interpreted unambiguously as a deficit or enhancement, relative to the appropriate genetic background for each mutant. One-way ANOVA (genotype) was performed on freezing scores at each time point for mice tested in the secondary screen and two-way ANOVA was performed (genotype by treatment) for conditioned taste aversion.

## Supporting Information

Text S1 Figure Legend for Supplemental Figure S1
(0.04 MB DOC)

Text S2 (0.05 MB DOC)

Text S3 Figure Legend for Supplemental Figure S2
(0.04 MB DOC)

Figure S1 Memory profiles of control animals in the primary screen.
(0.24 MB EPS)

Figure S2 Genetic background affects remote memory.
(0.01 MB TIF)

Table S1 (0.03 MB DOC)
