# Supplementary material for: A High Through-Put Reverse Genetic Screen Identifies Two Genes Involved in Remote Memory in Mice
Source: PLoS One. 2008 May 7;3(5):e2121. doi: 10.1371/journal.pone.0002121 (PMC2373872; doi:10.1371/journal.pone.0002121)
Supplement: Text S1 — Figure Legend for Supplemental Figure S1 (0.04 MB DOC) [file pone.0002121.s001.doc]

**Supplemental Figure 1.** Memory profiles of control animals in the primary screen. *a*) Remote Memory Deficit: Freezing and suppression ratios (SR) scores in this primary screen for the αCaMKII^+/-^ **hetero**zygous mice (white) compared to their wild-type controls (black) show that this mutation impairs only RM. *b*) Remote Memory Deficit: Freezing scores for C57BL/6J mice injected with 150mg/kg of anisomycin (Sigma) 30 minutes prior to training (white) show disruption of RM memory compared to saline injected controls (black). *c*) Hippocampus-dependency: Freezing scores for mice with dorsal hippocampal lesions (white) show that this lesion disrupts RM and STM context conditioning compared to sham-operated controls (black, see supplemental methods). *d*) Short-Term Memory Deficit: Freezing scores for the αCaMKII^-/-^ **homo**zygous mice (white) compared to their wild-type controls (black) show that this mutation impairs STM and consequently RM. BL = Baseline; IM = immediate memory; RM = 7 day memory; STM = short-term memory.
