# Supplementary material for: A High Through-Put Reverse Genetic Screen Identifies Two Genes Involved in Remote Memory in Mice
Source: PLoS One. 2008 May 7;3(5):e2121. doi: 10.1371/journal.pone.0002121 (PMC2373872; doi:10.1371/journal.pone.0002121)
Supplement: Text S2 — (0.05 MB DOC) [file pone.0002121.s002.doc]

**Supplemental Materials and Methods:**

**Surgery**

One day after training all animals were anesthetized with chloral hydrate (400mg/kg) and mounted in a stereotaxic apparatus (David Kopf Instruments, Tujunga, CA). The scalp was incised and retracted and the skull adjusted to place bregma and lambda in the same horizontal plane. Small burr holes were drilled in the skull for the placement of stainless steel electrodes (size 00 insect pin insulated with Epoxylite except for the 0.5mm tip). Mice were randomly assigned to either DH (dorsal hippocampus) or sham groups. DH mice received bilateral electrolytic lesions of the dorsal hippocampus by passing anodal current (3.0 mA for 5s) at two sites (AP –1.8, ML +/-2.0, DV –2.2). Sham mice were treated similarly but no current was passed. Histological verification of the lesion locations was performed at the end of behavioral testing.

**Back-crossing Itgb2^-/-^ and Soat1^-/-^ Mice**

Itg*b*2^-/-^ mice were crossed with wild type C57BL/6J mice from Jackson Laboratories (Bar Harbor, ME). Heterozygous offspring were crossed to generate wild type and homozygous deletion mutants. Genotyping was performed using qPCR. Soat1^-/-^ mice were crossed with C57BL/6NTac wild type mice from Taconic Farms (Germantown, NY). Heterozygous offspring were crossed to generate wild type and homozygous deletion mutants. Genotyping was performed using PCR.
