# Supplementary material for: A High Through-Put Reverse Genetic Screen Identifies Two Genes Involved in Remote Memory in Mice
Source: PLoS One. 2008 May 7;3(5):e2121. doi: 10.1371/journal.pone.0002121 (PMC2373872; doi:10.1371/journal.pone.0002121)
Supplement: Text S3 — Figure Legend for Supplemental Figure S2 (0.04 MB DOC) [file pone.0002121.s003.doc]

**Supplemental Figure 2.** Genetic background affects remote memory. *a*) Soat1^-/-^ mice (white) show normal memory at 7 days compared to wild type controls (black) when trained with 1 shock, (F_1,20_ = 0.403, p = 0.403; wt, n=11; mut, n=11). *b*) Soat1^-/-^ mice (white) show normal memory at 7 days compared to wild type controls (black) when trained with 3 shocks, (F_1,18_ = 0.029, p = 0.867; wt, n=10; mut, n=10). c) Itgb2^-/-^ mice (white) show normal memory at 7 days compared to wild type controls (black), (F_1,14_ = 3.72, p = 0.074; wt, n=8; mut, n=8).
