# Supplementary figures and images for: A High Through-Put Reverse Genetic Screen Identifies Two Genes Involved in Remote Memory in Mice
Source: PLoS One. 2008 May 7;3(5):e2121. doi: 10.1371/journal.pone.0002121 (PMC2373872; doi:10.1371/journal.pone.0002121)

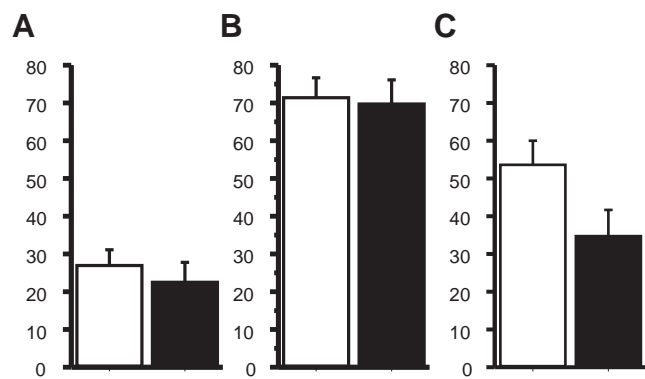

Supplement: Figure S2 — Genetic background affects remote memory. (0.01 MB TIF) [file pone.0002121.s005.pdf]
