# Supplementary material for: A High Through-Put Reverse Genetic Screen Identifies Two Genes Involved in Remote Memory in Mice
Source: PLoS One. 2008 May 7;3(5):e2121. doi: 10.1371/journal.pone.0002121 (PMC2373872; doi:10.1371/journal.pone.0002121)
Supplement: Table S1 — (0.03 MB DOC) [file pone.0002121.s006.doc]

Supplemental Table 1: Activity Levels of Putative Remote Memory Mutants

|  | **Wild-type** |  | Mutant |  |  |  |
| --- | --- | --- | --- | --- | --- | --- |
|  | **Activity** | n | **Activity** | n | F | p |
| Itgβ2 | 38.3 au | 80 | 48.9 au | 58 | (1,136)=44.1 | <0.0001 |
| Soat1 | 26.6 au | 49 | 33.0 au | 44 | (1,91)=8.44 | 0.0046 |
| Syn2 | 39.6 au | 44 | 40.0 au | 46 | (1,88)=0.014 | 0.91 |

*au=arbitrary units

**Supplemental Table 1.** Baseline activity levels for the three mutants tested in the secondary screen.
